# Single Atom Engineering for Nanorobotics

**DOI:** 10.1021/acsnano.4c06880

**Published:** 2024-07-24

**Authors:** Xiaohui Ju, Martin Pumera

**Affiliations:** † Future Energy and Innovation Laboratory, Central European Institute of Technology, Brno University of Technology, Purkyňova 123, 61200 Brno, Czech Republic; ‡ Advanced Nanorobots & Multiscale Robotics Laboratory, Faculty of Electrical Engineering and Computer Science, VSB−Technical University of Ostrava, 17 listopadu 2172/15, 708 00 Ostrava, Czech Republic

**Keywords:** nanorobotics, single-atom engineering, materials science

## Abstract

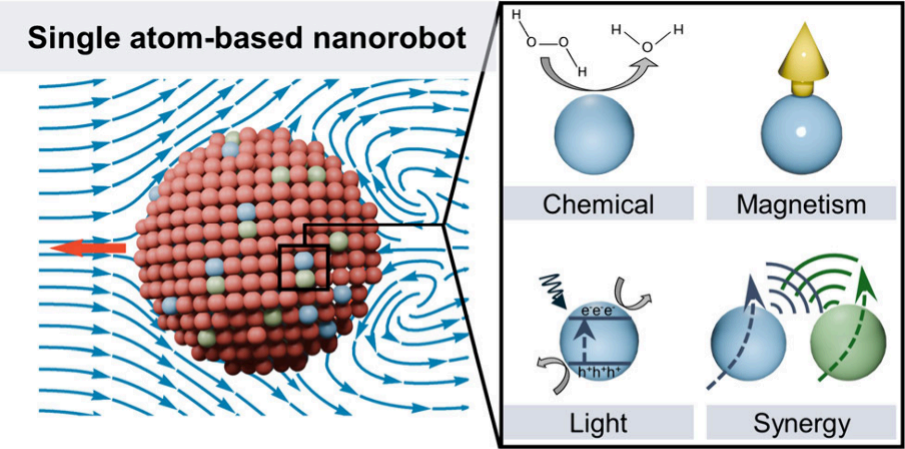

The fields of single atom engineering represent cutting-edge areas in nanotechnology and materials science, pushing the boundaries of how small we can go in engineering functional devices and materials. Nanorobots, or nanobots, are robotic systems scaled down to the nanometer level and designed to perform tasks at similarly small scales. Single atom engineering, on the other hand, involves manipulating individual atoms to create precise materials and devices with controlled properties and functionalities. By integrating single atom engineering into nanorobotics, we unlock the potential to enable the precise incorporation of multiple functionalities onto these minuscule machines with nanometer-level precision. In this perspective, we describe the nascent field of single atom engineering in nanorobotics.

Nanorobots are typically envisioned for use in medicine, environmental, food, and security applications.^1,2^ Nanorobots have the potential to revolutionize medical applications. For example, they can be engineered to target specific diseased cells, delivering drugs directly to the affected area maximizing therapeutic impact, performing surgery and diagnostics, as well as biofilm removal at hard-to-reach areas.^3−7^ Environmental applications of nanorobots include the detection and neutralization of pollutants or degradation of microplastics.^8^ In the field of the food industry, nanorobots find key applications in pollutant sensing and removal.^8,9^ In security applications, the sensing and degradation of nerve agents is the main application.^10^

Nanorobots themselves can be built similarly to LEGO blocks (Figure 1).^11^ One can start with an inert nanocarrier as chassis, to which one introduces a motion generator–the so-called nanomotor. The mechanism driving motion may involve catalytic chemical or photochemical conversion of a fuel, or the influence of an external physical field such as magnetism, light, electric field, or ultrasound. The next step is to introduce functionality, i.e., something the nanorobot should do, for example, to capture a biomolecule, decompose a pollutant, deliver a drug, perform an action, or kill bacteria. Furthermore, we can introduce taxis–navigation as directed by a chemical source, light, magnetic field, or gravity (so-called chemotaxis, phototaxis, magnetotaxis, and gravitaxis, respectively). The last step is the collective swarming behavior of many-body nanorobots. We can program the nanorobot by either physical or chemical programming, i.e., how the material of the robot behaves when exposed to certain physical or chemical stimuli.

**Figure 1:**
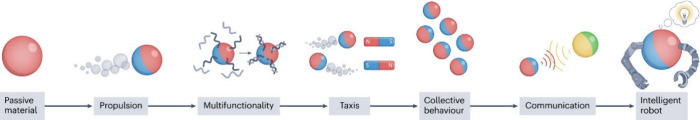
From passive materials to intelligent robots. Micro- and nanorobots are designed using micro- and nanomaterials and introducing: propulsion, the ability to move spontaneously by consuming a chemical fuel or under exposure to an external field; multifunctionality, the ability to perform multiple specific tasks; taxis, the adaptive response to environmental stimuli such as gradients of chemical species (chemotaxis), light (phototaxis), or magnetic fields (magnetotaxis); collective behavior, the cooperative action of robot ensembles to improve the efficacy of a process or to perform complicated tasks beyond an individual’s capability; communication, through which neighboring robots can operate in a coordinated and synchronized manner and exchange information. The scheme illustrates an example of a bubble-propelled Janus robot in which the red and blue hemispheres represent the structural-functional and engine sides, respectively. Adapted with permission from ref (11). Copyright 2023 Spring Nature.

The term “single atom engineering” was introduced about a decade ago.^12^ It builds on the idea that a single atom is the “ultimate nanoparticle” as it has “all the atoms on the surface” and distinct energy levels. Using an anchoring substrate, one can manipulate the energy levels of the orbitals of the separated individual atoms. Rather than investigating one single atom, mainstream single atom research aims to distribute individual single atoms in large quantities to see their macroscopic effect. Single atom engineering, the way the materials are fabricated and characterized, was discussed in many excellent review articles, which focused both on fundamentals^12,13^ as well as on applications, *e.g*., energy,^14^ sensing,^15,16^ or biomedicine;^3,17^ and it is not our aim to repeat them here.

Here we focus on the nascent field of single atom engineering in nanorobotics (Figure 2). Single atom-based nanorobots represent a revolutionary advancement over conventional counterparts due to their operational principles and unparalleled advantages across biological, environmental, and food applications. Unlike traditional nanorobots that rely on bulk nanomaterials, single atom-based nanorobots utilize individual atoms, enabling precise manipulation at the atomic scale. This capability enhances their functionality in biological contexts, where they can deliver drugs with pinpoint accuracy and interact precisely with intracellular structures. In environmental applications, their atomic precision enhances catalytic efficiency per unit mass, improving energy efficiency and effectiveness in tasks like pollution remediation. Furthermore, the reduced catalytic material loading minimizes environmental impact and biological toxicity. Their ability to integrate multiple functionalities within a single nanorobot configuration enhances versatility and applicability across various sectors.

**Figure 2:**
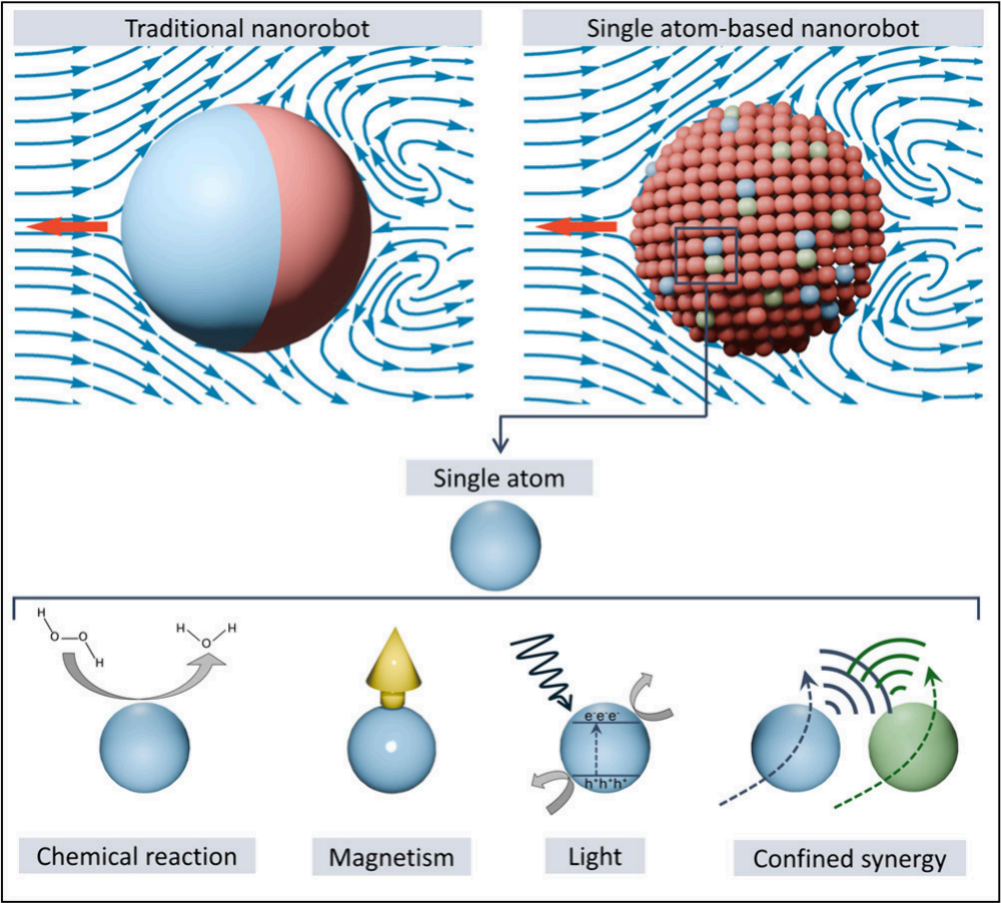
From conventional nanorobots to single atom-based nanorobots. Single atom-based nanorobots offer advantages, including atomic-resolution precision, high energy efficiency, reduced material toxicity for better compatibility, and multifunctionality within confined spaces. They can be engineered for propulsion using catalytic chemical reactions, single-atom magnetism, or light-induced photocatalysis. By integrating multiple species of single atoms, synergistic effects can be achieved, allowing for more efficient motion and cooperative behavior at a much smaller scale than conventional nanorobots.

Each step of the nanorobot fabrication and assembly process can benefit from the introduction of single atom engineering. The motoric part of nanorobots can greatly benefit from catalytically enhanced motion via single atom catalysis or from enzyme-mimicking reactions via single atom engineered material. The directional control of nanorobots known as taxis can be manipulated through the morphological and asymmetrical distribution of single atoms onto nanorobot chassis. The function of the nanorobot (or, if you want, its application) can benefit from single atom engineered surfaces generating reaction products, such as reactive oxygen species (ROS), to perform functions such as biofilm eradication,^18^ microplastics degradation,^19^ and cancer treatment.^20,21^ It is not surprising that efforts to modify the materials from which nanorobots are fabricated have been carried out in the past decade. In the process of doping, small concentrations of doping atoms (*i.e*., silver on ZnO_2_^22^ or gadolinium on SiO_2_ particles^21^) are used although no detailed characterization and confirmation of single atom doping (that is, with well-dispersed atoms on the structure) has been performed.^7^ Such Ag-doped ZnO_2_ functions both to enhance photoelectrochemical propulsion as well as bacterial film eradication.

Given that the motion of nano- and microrobots is fundamental to their functionality, we will initially demonstrate how single atom engineering can effectively alter and enhance the motion of these minuscule devices. Taking chemical propulsion as an example, downsizing catalyst layers in tens of nanometers to single atom level greatly enhances the catalytic efficiency while minimizing the total metal loading. Iron (Fe) based single atom catalysts can be densely anchored onto hierarchically porous graphitic carbon materials via pulsing H_2_-pyrolysis approach (Figure 3a).^23^ The resulting Fe single atom catalysts (SACs) have excellent catalytic activity for hydrogen peroxide decomposition into oxygen, leading to efficient propulsion due to the bubble generation in combination with the asymmetrical shape of the substrate. Comparing the Fe SAC nanorobots with the most used H_2_O_2_-fuel nanomotors, it exhibited much higher catalytic efficiency, further enhancing its mobility.

**Figure 3:**
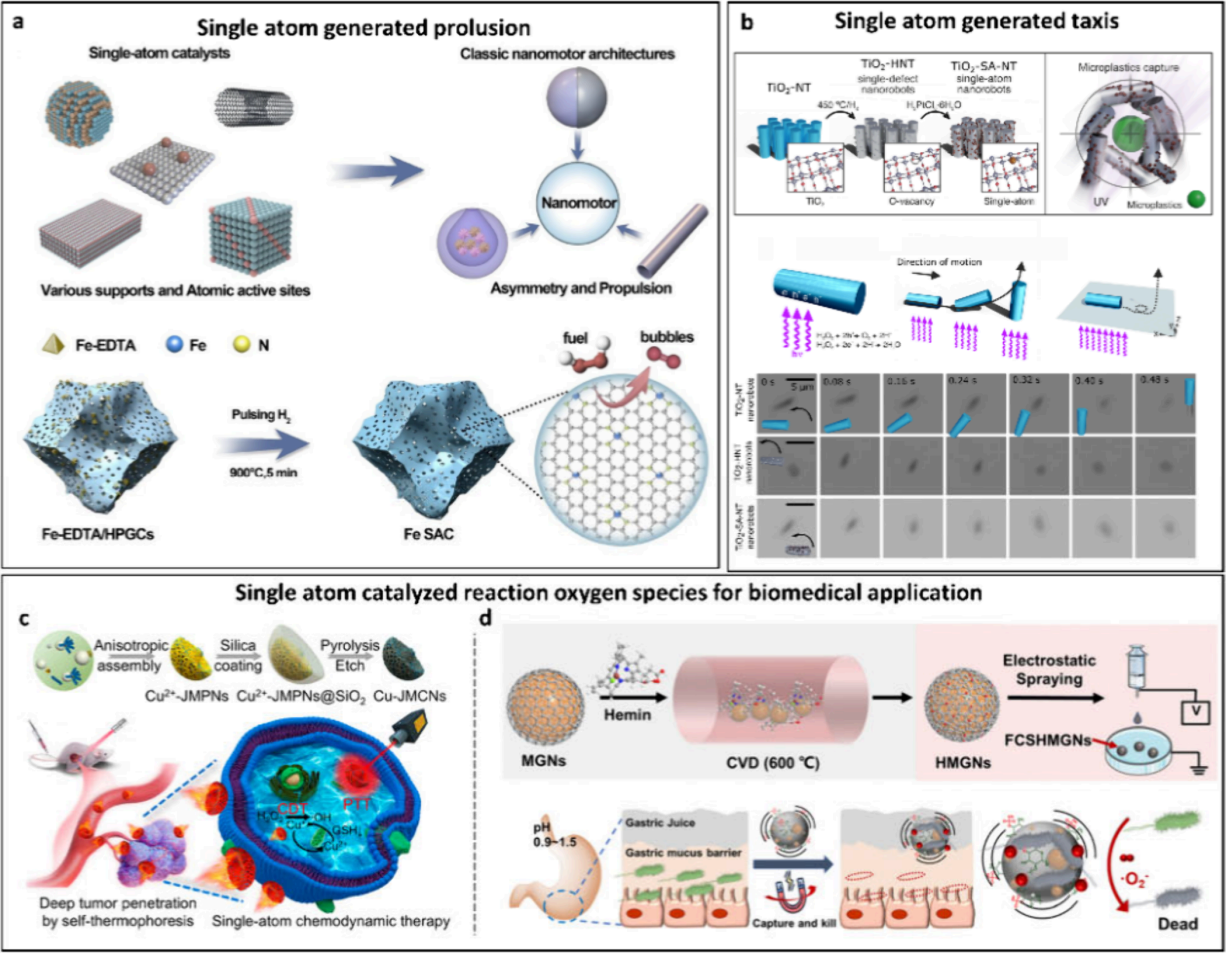
Single atom engineering of nanorobots generating motion, taxis, and multifunction. (a) Schematic illustration of SACs anchored to support with different dimensions compared to classical nanomotors. Example of fabrication of Fe SAC for generating bubbles based on chemical propulsion. Adapted with permission from ref (23). Copyright 2024 Elsevier. (b) Schematic illustration of the example using single atoms directional navigation. The scheme illustrates the fabrication of TiO_2_-based tubular nanorobots toward Pt single-atom anchored tubular nanorobots for the capture of microplastics under UV irradiation. These SAC tubular nanomotors exhibit negative photogravitaxis through a stand-up motion mechanism. Adapted with permission from ref (19). Copyright 2024 Wiley-VCH. (c) Single-atom Cu-engineered jellyfish nanomotors powered by near-infrared light (NIR). Active movement of the nanomotors enhanced the penetration of tumors due to self-thermophoretic diffusion, and Cu single atoms generated sufficient reactive oxygen species for chemodynamic therapy for tumor inhibition. Adapted with permission from ref (20). Copyright 2023 American Chemical Society. (d) Single atom engineered microsweepers for the eradication of *Helicobacter pylori* in the human stomach. Diagram illustrating the preparation of iron single-atom anchored materials on the graphitic shell and magnetic core of the robot Fe SACs exhibit enzyme mimicking ROS-generating activity. Local magnetic field powered the motion of these nanorobots with the functionality of capturing and destroying *H. pylori* in acidic stomach environment. Adapted with permission from ref (24). Copyright 2023 Royal Society of Chemistry.

Single atom engineering on nanorobots not only enhances the propulsion but can also be applied as an approach to generate navigation and taxis behavior. The manipulation of point defects and the addition of individual platinum (Pt) atoms and atomic-scale Pt elements to titanium dioxide (TiO_2_)-based nanotubes (refer to Figure 3b) was explored to enhance the motility and directional control of cylindrical nanorobots.^19^ This single atom engineering approach was investigated to assess its influence on nanorobot mobility. The creation of point defects in the nanotubes was achieved by heating the TiO_2_ structures in a hydrogen-rich environment. Following this, Pt atoms at the atomic scale were inserted into the nanotubes’ structure through a wet-chemical deposition method, which built on the previously established point defect engineering process. These nanorobots harnessed light energy to power their motion. When exposed to ultraviolet (UV) light, the SAC-based nanorobots exhibited a type of movement away from the light source, called negative photogravitaxis, demonstrating the nanorobot’s directional control. It is due to the asymmetric distribution of the single atoms onto the substrate generating asymmetrical phoretic distribution, further maneuvered the nanorobot to perform a “stand-up” motion. These SACs-based nanorobots have demonstrated significant potential in environmental applications, particularly in the capture of microplastics. These single atom-based nanorobots create asymmetrical local electric fields through ionic diffusiophoresis. This phenomenon influences nearby passive microplastics, leading to their spontaneous capture. The opposite surface charges of these nanorobots enable them to attract microplastics electrostatically, which is further enhanced by their motion “on the fly”.

Biomedical nanorobots can also benefit from the functionality generated through crafting single atoms onto the available nanomotors. Doping single-atom copper (Cu) onto jellyfish-like mesoporous carbon nanomotors, these biomedical nanorobots demonstrated enhanced tumor penetration tumor inhibition. As shown in Figure 3c, these motors are powered by near-infrared light (NIR) generating enhanced self-propulsion due to asymmetrical self-thermophoresis. Cu single atoms can catalyze H_2_O_2_ into toxic reactive oxygen species for chemodynamic therapy.^20^ Combined with improved cellular uptake and penetration of *in vivo* tumors, this design provides a possibility for integration of single atom catalysis with autonomous nanomotor for active nanomedicine. A similar approach builds the nanomotor chassis by employing magnetic nanoparticles encased in a graphitic shell as their core structure (Figure 3d). An external magnetic field was utilized to impart motion functionality to these microrobots. Single-atom iron (Fe) anchored on the graphitic shell of the micromotors function as pH-responsive oxidase-like nanozymes. These single atoms facilitated ROS-generating catalysis in acidic conditions to aid the microrobots in eradicating *Helicobacter pylori* in the human stomach. These biomedical applications included bacterial eradication in stomach conditions.^24^

Nanorobots and single atom engineering are exciting fields that epitomize the spirit of innovation in nanotechnology and materials science. As research progresses, we can anticipate breakthroughs that will translate these high-concept technologies into practical tools, significantly impacting medicine, environmental science, and manufacturing. Both fields face significant challenges. For nanorobots, issues such as power supply, navigation, functionality, and biocompatibility are always a challenge. The scale of operation makes traditional power sources impractical while navigation inside complex environments like the human body requires sophisticated control strategies. For single atom engineering, the main challenges lie in toxicity, scalability, and stability. Reducing catalytic nanoparticles to single atoms decreases their toxicity for biomedical applications, owing to lower metal loadings.^25^ Real-time optical imaging like chemiluminescence, second near-infrared light, and photoacoustic imaging are essential for evaluating the biosafety of SAC nanorobots, spanning from cellular uptake to systematic toxicity evaluation. Developing biodegradable materials can address safety concerns. Stability issues, influenced by environmental triggers that lead to metal leaching, highlight the criticality of preserving isolated active sites. The challenge of scalable synthesis lies in balancing the high loading of catalysts with maintaining single-atomic dispersion, requiring advancements in optimizing SAC synthesis methods.

There are many opportunities for single atom engineering to advance nanorobotics. The motion of the nano- and microrobots can be enhanced by various single atom dopants, efficiently catalyzing chemical fuel (or water) decomposition and powering nanorobots. Nanozymes crafted through single atom engineering offer a stable alternative to conventional enzymes, empowering micro- and nanorobots to survive more harsh conditions. By designing single atom magnetic domains, the field of magnetically powered and navigated nanorobots can move forward. Furthermore, single atom engineering can facilitate the fabrication of nanorobots of truly nanometer size; so far, the smallest nanorobots were of size over 10 nm to incorporate all the LEGO components. Such nanorobots would be smaller than most enzymes. Single atom engineering can benefit from coupling several types of single atoms, each having a specific role, or working with other atom catalytic sites in tandem. Dual atom engineering or precision metal cluster engineering serves as an extension of single atom engineering to reach more precision confinement. Nanoarchitectonics principles for precise control of single atom position are envisioned to be employed in the future.^26^ The applications of single atom functionalities are endless. We might consider the visionary prospect of single atom engineered nanorobots supplanting antibiotics in infection treatment, orchestrating swarms of these tiny marvels to execute nanosurgeries, or spearheading the degradation of nanoplastics—an exciting frontier in advanced medical and environmental interventions.
